# Genome-Wide Gene Expression Analyses of *BRCA1*- and *BRCA2*-Associated Breast and Ovarian Tumours

**DOI:** 10.3390/cancers12103015

**Published:** 2020-10-16

**Authors:** George A. R. Wiggins, Logan C. Walker, John F. Pearson

**Affiliations:** 1 Mackenzie Cancer Research Group, Department of Pathology and Biomedical Science, University of Otago, Christchurch 8011, New Zealand; george.wiggins@otago.ac.nz; 2 Biostatistics and Computational Biology Unit, Department of Pathology, University of Otago, Christchurch 8011, New Zealand; john.pearson@otago.ac.nz

**Keywords:** breast cancer, ovarian cancer, microarray, gene expression, familial cancer

## Abstract

**Simple Summary:**

Variants in the breast cancer susceptibility genes BRCA1 and BRCA2 increase the risk of developing breast and ovarian cancers. Over the past two decades researchers have aimed to identify gene expression changes associated with high-risk BRCA1 and BRCA2 variants. In this review we explore the replicability of BRCA1- and BRCA2-associated gene expression profiles in diseased and normal tissue. We highlight the impact of experimental factors and study designs on the comparability and utility of gene expression profiles associated with high-risk BRCA1 and BRCA2 variants. Additionally, we emphasise the importance of controlling for confounding molecular features that may influence the design of study cohort groups.

**Abstract:**

Germline pathogenic variants in *BRCA1* and *BRCA2* increase cumulative lifetime risk up to 75% for breast cancer and 76% for ovarian cancer. Genetic testing for *BRCA1* and *BRCA2* pathogenic variants has become an important part of clinical practice for cancer risk assessment and for reducing individual risk of developing cancer. Genetic testing can produce three outcomes: positive (a pathogenic variant), uninformative (no pathogenic variant) and uncertain significance (a variant of unknown clinical significance). More than one third of *BRCA1* and *BRCA2* variants identified have been classified as variants of uncertain significance, presenting a challenge for clinicians. To address this important clinical challenge, a number of studies have been undertaken to establish a gene expression phenotype for pathogenic *BRCA1* and *BRCA2* variant carriers in several diseased and normal tissues. However, the consistency of gene expression phenotypes described in studies has been poor. To determine if gene expression analysis has been a successful approach for variant classification, we describe the design and comparability of 23 published gene expression studies that have profiled cells from *BRCA1* and *BRCA2* pathogenic variant carriers. We show the impact of advancements in expression-based technologies, the importance of developing larger study cohorts and the necessity to better understand variables affecting gene expression profiles across different tissue types.

## 1. Introduction

Germline pathogenic variants in the tumour-suppressor genes, *BRCA1* and *BRCA2*, predispose humans to breast and ovarian cancer with reduced effects on the risk of cancer in other tissues. Up to 1 in 200 individuals in the population carry a pathogenic variant in these genes [1] which explains approximately 5% of breast cancers [2] and over 10% high-grade serous ovarian cancers [3]. For pathogenic variants in *BRCA1*, the cumulative risk by age 70 years is 44–75% for breast cancer and 43–76% for ovarian cancer [4]. For pathogenic *BRCA2* carriers, the respective risks are 41–70% for breast cancer and 7.5–34% for ovarian cancer [4]. Predictive genetic testing can identify individuals who carry variants in *BRCA1* and *BRCA2* which confer risk and are therefore of clinical importance. Such information can help clinicians with developing strategies for treatment and the prevention of disease.

Genetic testing for *BRCA1* and *BRCA2* can produce three possible results: positive (a pathogenic variant is identified), uninformative (no pathogenic variant) or a variant of uncertain significance. Variants of uncertain significance are typically rare alterations to the gene sequence that have been assessed for association with cancer phenotype/s but risk association remains uncertain [5]. More than one third of known *BRCA1* and *BRCA2* variants are reported as variants of uncertain significance (https://www.ncbi.nlm.nih.gov/clinvar/ accessed March 2020), presenting a challenge for the genetic counsellor and their patients.

Informed clinical decision-making that is based on genetic test results requires the implementation of reliable variant classifications. Laboratory-based methods that can distinguish between carriers of known pathogenic variants and non-carriers have utility for the classification of sequence variants of unknown clinical significance. There are now many studies demonstrating the association of gene expression profiles from tumour- and stromal-derived cells with different breast cancer subtypes (Table 1). Distinct patterns of gene expression have also been explored in different cell types from individuals with and without *BRCA1* and *BRCA2* pathogenic variants. However, despite efforts to identify BRCA-associated gene expression signatures, the level of consensus between studies is unclear. It therefore remains uncertain whether gene expression analysis has been a successful approach for variant classification and whether results could translate into a diagnostic setting. Here we address these issues by reviewing reported gene expression profiles from *BRCA1* and *BRCA2* pathogenic variant carriers, and consider the impact of the design of studies on their comparability.

## 2. *BRCA1*- and *BRCA2*-Associated Breast Tumours

We reviewed a total of nine studies (Table 1) that assessed differences in gene expression profiles between breast tumours from *BRCA1* pathogenic variant carriers and non-*BRCA1* pathogenic variant carriers. Four of these also assessed differences between *BRCA2* and non-*BRCA2* pathogenic variant carriers.

The first study that attempted to identify a BRCA-like expression phenotype quantified the expression of 5000 probes across 21 breast tumours [6]. The expression of 51 genes was able to accurately segregate the three tumour genotypes (*BRCA1*, *BRCA2* and sporadic). Furthermore, 9 genes were associated with *BRCA1*-related breast tumours and 11 genes were associated with *BRCA2*-related breast tumours. The nine genes associated with *BRCA1* carriers accurately classified 95% (21/22) of breast tumours. Similarly, the 11-gene *BRCA2*-related list correctly classified 82% (18/22) of tumours. The only misclassified tumour using the *BRCA1* classifier had *BRCA1* promoter hypermethylation and reduced *BRCA1* expression, suggesting tumours without a pathogenic variant may display a “BRCA-like” phenotype. As the classifications were performed on the same samples used to generate the classifier, any interpretation of accuracy should be made cautiously. In an independent dataset, the 51 genes were not able to segregate *BRCA1*-associated tumours from sporadic tumours [11]. The dataset generated by Dudaladava and colleagues only identified 40 of the 51 genes described by the Hedenfalk study. Additionally, a major confounder of the Hedenfalk study was the oestrogen receptor (ER) status of the samples selected [6]. All the *BRCA1*-associated tumours were ER negative, compared with 33% of the non-*BRCA1*-associated tumours. ER status has since been revealed as a major driver of gene expression differences, irrespective of genotypes [8,19]. Therefore, the lack of classification accuracy in the Dudaladava study may be due to tumour phenotype, as all tumours were ER negative in this study or, alternatively, the strength of the 51 gene classifiers may hinge on the 11 absent genes.

In an attempt to account for *BRCA1*-associated tumour phenotypes, investigators have explored *BRCA1*-associated gene expression profiles in hormonal negative tumours [8,11,12]. van’t Veer and colleagues optimised a 100 gene *BRCA1* classifier by exploring gene expression profiles of 38 ER-negative breast tumours [8]. The 100 gene classifier accurately classified 95% (36/38) of ER-negative breast tumours. Similarly to Hedenfalk et al., one tumour misclassified as a *BRCA1*-associated tumour was reported to have hypermethylation of the *BRCA1* promoter. Martin and colleagues [12] utilised the data generated by van’t Veer et al., and investigated genes involved in BRCA1-related functions. Three genes (*RAD51, RAD54* and *RAD51AP1*) involved in homologous recombination were differentially expressed in *BRCA1*-associated ER-negative tumours. Despite using the same dataset, none of these three genes were present in van’t Veer’s 100 gene classifier. Dudaladava et al. [11] selected the 100 most differentially expressed *BRCA1*-associated genes. Unfortunately, as significance was not provided it is difficult to interpret the likelihood of a real difference between tumour types. Nevertheless, these 100 genes were able to accurately cluster 93% (13/14) of breast tumours based on *BRCA1* variant status.

The development of breast tumour intrinsic subtype predictions allowed investigators to interrogate tumour based on subtype [18,19,23,30,31]. Similar to ER status, subtype has a major influence on gene expression profiles [18,19]. Lisowska and colleagues were able to identify 423 genes significantly (*p* < 0.001) differentially expressed in *BRCA1*-associated tumours. The 423 genes were a poor predictor for variant status, only correctly classifying 69% of tumours in the training dataset. Similarly, the 423 genes poorly classified the 21 tumours from the Hedenfalk et al., study, with only 64% correctly classified [19]. Gene expression profiles from *BRCA1*-associated tumours were observed to be more similar to sporadic breast tumours than non-*BRCA1/2* (BRCAx) hereditary breast tumours. The similarity of some sporadic tumours to *BRCA1*-associated tumours is consistent with those observations in tumours with hypermethylated *BRCA1* promoters described in early studies [6,8]. One large study (*n* = 577) combined tumours containing hypermethylated *BRCA1* promoters with *BRCA1* carriers [20]. *BRCA1*-impaired (pathogenic carriers and hypermethylated *BRCA1* promoter) tumours had 321 genes differentially expressed from basal-like sporadic tumours. Unfortunately, we only identified four genes (*RB1*, *BRCA1*, *CDK6* and *CCDN1*) in the manuscript, making interpretation of their findings difficult.

Three studies employed well-utilised expression arrays (Illumina and Affymetrix) which consisted of approximately 40,000 probes targeting approximately 25,000 RefSeq annotated genes [18,23,31]. Waddell et al. [18] assessed familial breast tumours from 75 patients who carried pathogenic variants in *BRCA1* or *BRCA2*, or were BRCAx, to determine molecular heterogeneity between and within each subgroup. A total of 277 genes were differentially expressed between *BRCA1* and BRCAx tumours, and 31 genes were differentially expressed between *BRCA2* pathogenic variants and BRCAx tumours. The difference observed in the size of the gene lists suggested that BRCAx tumours are more similar to tumours from *BRCA2* pathogenic variant carriers than *BRCA1* pathogenic variant carriers. This hypothesis is reinforced by the intrinsic subtypes of *BRCA1*, *BRCA2* and BRCAx tumours. *BRCA1* carriers were largely (74%) basal-like, while tumours from *BRCA2* carriers and BRCAx were largely (73% and 60%) luminal, an observation which has also been observed in subsequent studies [20,23,25]. Larsen and colleagues [23] studied 183 breast tumours (33 *BRCA1*, 22 *BRCA2*) and developed a 110-probe signature to classify *BRCA1* breast tumour within the basal-like subtype with an accuracy of 83% (sensitivity: 82%, specificity 85%). Similarly, a 100-probe signature was identified for classification of luminal B *BRCA2* carriers with an accuracy of 89% (sensitivity: 88%; specificity: 90%).

A meta-analysis using four published datasets [18,25,31,32] was conducted to identify differentially expressed genes between different *BRCA1/2* pathogenic variant carriers and BRCAx/sporadic breast tumours [29]. Two hundred and seventy eight genes were consistently differentially expressed (*p* < 0.05, 0.6 < *log*2FC < −0.6) amongst the four studies. Differentially expressed genes were mapped to transcription factor binding sites to identify key regulators, including five transcription factors (*FOXM1, TFAP2C, FOXA1, ESR1* and *GATA3*) that were differentially expressed (*p* < 0.05, FC *>* 1.5) in at least three studies.

Table 2 highlights the poor overlap between studies of gene expression profiles in breast tumours. The largest overlap is seen between analyses performed by Waddell et al., and Akbari et al. [18,29]. However, the overlap may be driven by the Akbari et al., using data generated by Waddell et al. Furthermore, each of these analyses produced the greatest amount of genes associated with *BRCA1* or *BRCA2* pathogenic status. Only one gene, *TOB1*, was identified as associated with *BRCA1* carrier status by more than two studies [6,11,19].

## 3. *BRCA1*- and *BRCA2*-Associated Ovarian Tumours

Additional to breast tumours, three studies have been published (Table 1) that assessed differences in gene expression profiles between *BRCA1/2*-associated ovarian tumours and BRCAx ovarian tumours [7,17,22], and a further study identified a precancerous signature in the fallopian tube tissue of the *BRCA1* pathogenic variant [16]. Jazaeri and colleagues quantified gene expression for 61 ovarian tumours, which included 18 *BRCA1*- and 16 *BRCA2*-associated tumours [7]. The expression of all approximately 7500 probes was able to distinguish *BRCA1*-associated tumours from *BRCA2*-associated tumours. However, the sporadic ovarian tumours were mixed amongst these two populations, reinforcing the observation that a potential “BRCA-like” expression profile may exist in a subset of sporadic tumours previously described in breast tumour analyses [6,8,19]. In addition, only 9 and 3 genes were differentially expressed between sporadic tumours and *BRCA1*- and *BRCA2*-associated tumours, respectively. By comparison, 110 genes were differentially expressed genes between *BRCA1*- and *BRCA2*-associated ovarian tumours. Interestingly, in a later study, George et al. [22] were unable to replicate the tumour clustering despite performing the analysis on the same dataset, thus implicating that these results may be a false positive.

The Jazaeri dataset was analysed by Konstantinopoulos et al., and a 60-gene “BRCA-like” signature was identified using a selective sample exclusion criteria [17]. Samples were excluded based on transcriptome-wide unsupervised clustering which generated three distinct clusters termed “sporadic”, “BRCA1” and “BRCA2”. In an attempt to purify these groups, samples that were misclassified were excluded from further analysis, leaving only 23 familial (13 *BRCA1*, 10 *BRCA2*) and 14 sporadic ovarian tumour samples. These 37 samples were used to identify a 60-gene signature that accurately classified (94%) the selected BRCA-associated ovarian tumours from sporadic tumours. As the accuracy was only tested on the training dataset it is difficult to determine the utility of a 60-gene classifier. Furthermore, the highly selective exclusion criteria implemented limits the translation of the classifier to any real world clinical sample sets.

George et al., used three public datasets [7,33,34] to identify differences between ovarian tumours defective for *BRCA1* and *BRCA2* compared to sporadic tumours [22]. Expression data from The Cancer Genome Atlas (TCGA) [33] network was used to identify genes differentially expressed that correlated with *BRCA1* and *BRCA2* mutation status (somatic or germline variants). A total of 65 genes were differentially expressed between *BRCA1/2*-mutated and non-mutated ovarian tumours, and 34 genes were differentially expressed between *BRCA1*-mutated and non-BRCA-mutated ovarian tumours. No genes were differentially expressed between *BRCA2*-mutated and non-BRCA-mutated ovarian tumours. In addition to identifying differentially expressed genes, George et al., investigated the discriminatory power of Konstantinopoulos’ 60-gene “BRCA-like” classifier [17]. Three independent datasets [7,33,34] were used to compare classifiers from George et al., and Konstantinopoulos et al. All three cohorts showed that the TCGA-derived classifiers outperformed the previously published 60-gene “BRCA-like” signature with area under the receiver operating characteristic (ROC) curves ranging from 0.77–0.89 versus 0.55–0.63, respectively.

To assess early tumorigenic events, Press et al., conducted a study of noncancerous fallopian tube (with and without *BRCA1* pathogenic variants) and ovarian carcinomas from individuals with *BRCA1* pathogenic variants [16]. This study identified 152 probes differentially expressed (FC *>* 1.8 and *p* < 0.01) between noncancerous *BRCA1* and BRCAx fallopian tubes. A further 4079 probes were differentially expressed between noncancerous fallopian tubes from BRCAx and *BRCA1*-associated ovarian tumours. A preneoplastic signature was defined by the 41 probes differentially expressed in the same direction across each analysis [16]. Unsupervised hierarchical clustering was performed with an additional 12 normal fallopian tube samples with *BRCA1* pathogenic variants. Interestingly, five samples clustered with *BRCA1*-associated tumours (fallopian or ovarian), the remaining seven clustered with normal fallopian tubes with no pathogenic variant. These results suggest the limitation of implementing tumour-derived classifiers to predict variant status in normal tissue. Similar to that seen in breast tissue, there was overwhelmingly poor overlap of genes between studies (Table 1). Pairwise comparison saw no more than two genes in common between *BRCA1/2*-associated ovarian studies.

## 4. Non-Tumour Tissue from *BRCA1* and *BRCA2* Pathogenic Variant Carriers

To assess gene expression patterns of BRCA-variant carriers, studies have also investigated stromal noncancerous tissue, lymphoblastoid cell lines (LCLs), lymphocytes and peripheral blood mononuclear cells (Table 1).

### 4.1. Fibroblasts

Under the hypothesis that DNA repair is impaired in *BRCA1/2* pathogenic variant carriers, two studies explored induced DNA damage in short-term cultures of fibroblasts after γ-irradiation. Kote-Jarai and colleagues established short-term fibroblast cultures from 14 women (9 *BRCA1* pathogenic variant carriers) who underwent prophylactic mastectomy or breast reductive surgery [9]. Using two independent methods involving differential expression and class prediction, 122 *BRCA1*-associated genes were identified, of which 79 were identified by both methods. Genes differentially expressed accurately clustered *BRCA1*-associated fibroblasts and only one sample was misclassified as wild-type *BRCA1*.

A subsequent study utilised skin biopsies of 30 (10 *BRCA1*, 10 *BRCA2* and 10 sporadic) women with a history of breast cancer who were disease free at time of recruitment [10]. The top 200 genes were identified that best discriminated between each genotype post *γ*-irradiation. Thus, three gene panels were developed (*BRCA1* vs. BRCAx, *BRCA2* vs. BRCAx and *BRCA1* vs. *BRCA2*). The three gene panels accurately clustered all samples based on *BRCA1/2* genotype. However, hierarchical clustering was performed on the training dataset and no inclusion of the third genotype was tested. Therefore, the accuracy and robustness of the genes’ discriminative power was not examined. Furthermore, each of the studies by Kote-Jarai et al. [9,10] has poor overlap in discriminative genes, with only four genes (*ADNP*, *CDKN1B*, *FYN* and *SPIN*) in common. One explanation for the lack of concordance may be due to sample type. In the later study, normal breast tissue was acquired from women post-cancer and post-treatment. It is plausible that the development and treatment of breast cancer may have altered the expression profile of local normal breast tissue.

### 4.2. Lymphoblastoid Cell Lines (LCLs)

Eight studies investigated the effect of *BRCA1/2* pathogenic variants on gene expression in immortalised LCLs [13,15,24,26] or peripheral blood [14,21,27,28]. Waddell et al. [13] assessed the effects of missense and truncating *BRCA1/2* pathogenic variants post γ-irradiation in 78 LCLs (23 *BRCA1*, 22 *BRCA2*, 27 BRCAx). Compared to missense pathogenic variants, truncating pathogenic variants had a larger effect on expression profiles. Truncating *BRCA1* pathogenic variant carriers had 2474 genes differentially expressed compared with BRCAx cases, and missense *BRCA1* pathogenic variant carriers only had 599 genes differentially expressed. Similarly for *BRCA2* pathogenic variant carriers, 3932 genes were differentially expressed when assessing truncating variants and 788 genes were differentially expressed when assessing missense variants. The top 200 genes (ranked by *p*-Value) of each gene list were used to predict the pathogenic variant status of LCLs. Only the details of these 200 genes were made available in the published report. For the two *BRCA1* gene lists (truncating and missense), the truncating variant classifier was most accurate, correctly predicting the mutation status in 76% (38/50) of the LCLs. In comparison, both the truncating and missense *BRCA2* classifiers correctly predicted 73% of the LCLs. However, the classifier derived from *BRCA2* truncating variants misclassified all *BRCA2* missense-associated LCLs. It is unclear whether inclusion of all differentially expressed genes would improve accuracy.

Walker et al. [15] used a pooled-RNA strategy to assess 27 LCLs derived from affected women in high-risk breast cancer families (9 *BRCA1*, 9 *BRCA2* pathogenic variant carriers and 9 BRCAx) and 9 LCLs from healthy individuals, before and after treatment with mitomycin C. This study identified 36 genes which overlapped three different analyses that compared samples based on: (1) BRCA variant status, (2) mitomycin C treatment status and (3) disease status. A classifier was built using the expression profile of nine RT-qPCR validated genes which distinguished *BRCA1* from *BRCA2* variant carriers with 83% accuracy. However, inclusion of all *BRCA1, BRCA2* and BRCAx LCLs decreased the performance with a maximum of 59% prediction accuracy.

Fielotter et al. [24] compared the expression profiles of 31 *BRCA1*-associated LCLs against 38 control LCLs post *γ*-irradiation. Genes were identified based on the predictive value of classifying *BRCA1* status. Interestingly, the authors used raw microarray data along with quantile normalised expression data to identify candidate genes. The analysis of raw intensity values may introduce biases especially with fluctuations in sample RNA load. In total, 43 genes were identified that best classified the 53 samples used as a training set. All 16 test samples were correctly classified and in total the gene set performed accurately (sensitivity = 84%, specificity = 92%). However, only 3/43 genes were validated by RT-qPCR.

Pouliot et al. [26] investigated gene expression profiles in LCLs from 117 women with (affected) and without breast cancer (unaffected) from related individuals with or without pathogenic *BRCA1* or *BRCA2* variants. Ninety-five transcripts were differentially expressed between unaffected BRCAx LCLs and either *BRCA1* pathogenic variants, *BRCA2* pathogenic variants or affected BRCAx. These 95 transcripts segregated *BRCA1* and *BRCA2* pathogenic variants from BRCAx LCLs; however, they were not able to discriminate each variant type. Post-hoc analysis suggested that 69 transcripts were differentially expressed between LCLs with *BRCA1* pathogenic variants and unaffected BRCAx LCLs, and 71 transcripts were differentially expressed between LCLs with *BRCA2* pathogenic variants and unaffected BRCAx LCLs.

### 4.3. Peripheral Blood

Expression profiles of 30 peripheral blood samples identified 133 differentially expressed genes associated with *BRCA1* pathogenic variants [14]. However, after adjusting for multiple testing no genes were statistically significant. The 133 genes were able to accurately classify 80% (11/15) of the *BRCA1* pathogenic variants and 100% (15/15) of the non-variants. Again, the classification was only performed on the training dataset and not validated in an independent cohort.

Salmon and colleagues used peripheral blood (lymphocytes) from 80 individuals, including *BRCA1* (*n* = 13) and *BRCA2* (*n* = 10) pathogenic variant carriers [21]. Analysis of *γ*-irradiated lymphocytes from *BRCA1* pathogenic variants revealed 137 probes that were differentially expressed compared to control lymphocytes. Interestingly, a greater effect was observed in *BRCA2*-associated lymphocytes with 1345 probes differentially expressed compared to controls. In an attempt to select the most discriminate genes, only genes with fold changes greater than two and consistent expression patterns across all samples were considered. Thirty-six genes met this criteria, the majority of which were enriched for transcription and DNA binding processes. Furthermore, RT-qPCR was used to test the accuracy of the classification and to refine these discriminatory genes. Firstly, of the 36 genes, 21 showed significant differences (measured by RT-qPCR) associated with *BRCA1* or *BRCA2* status. The classifier was further refined by ROC curve analysis of each gene. ROC curve analysis concluded that three genes performed poorly and these were excluded from the classifier. The remaining 18 genes accurately classified lymphocytes based on *BRCA1* and *BRCA2* status in an independent cohort of 57 individuals. In contrast to findings in irradiated lymphocytes, non-irradiated lymphocytes displayed a greater change in expression profiles for carriers of pathogenic *BRCA1* variants compared to *BRCA2* variants [27]. Compared to wild-type controls, 203 and 29 genes were differentially expressed associated with *BRCA1* and *BRCA2* pathogenic variants. The discrepancy between irradiated and non-irradiated lymphocytes and variant status may be due to differential dependency of *BRCA1* and *BRCA2* and the response to *γ*-irradiation. In addition, immortalised LCLs *BRCA1* but not *BRCA2*-associated LCLs are sensitive to γ-irradiation [35].

Due to the association of BRCA1 and BRCA2 proteins with telomere maintenance [36], peripheral blood was collected from 40 women (31 with breast cancer) and telomere-associated gene expression levels were assessed [28]. *BRCA1* pathogenic variants had a greater disruption on telomere-associated genes. Forty-six and eight genes were differentially expressed between *BRCA1* and *BRCA2* carriers, respectively. Despite greater disruption to the expression of telomere-associated genes, there was no difference in telomere length between *BRCA1* and *BRCA2* carriers.

## 5. Reproducibility between Expression Studies

We found a poor overlap of *BRCA1*/2 associated genes between expression studies, irrespective of tissue type (Table 2). The majority of studies identified a high number of differentially expressed genes compared to sample sizes, and used these to produce classifiers. The lack of agreement between studies is consistent with the classifiers over-fitting the dataset. Furthermore, lack of consensus may be due to other factors, including differences in study design and statistical approaches undertaken.

### 5.1. Sample Selection

Differences in sample selection can be summarised as three broad ideas: (1) definitions of experimental and control groups, (2) tumour matching and (3) purity of tumour samples.

Control arms can broadly be split into two categories: unselected (e.g., sporadic cancer) or BRCAx individuals. BRCAx and sporadic tumours themselves are heterogeneous and generate distinct gene expression profiles [37]. Fernàndez-Ramires et al., identified two BRCAx subgroups and two sporadic subgroups based on gene expression profiling. Although there was some overlap between the BRCAx and sporadic subgroups, results suggest that there are sub-populations of tumours that differ within each control group. Therefore, comparison of *BRCA1* or *BRCA2* variant carriers would be expected to exhibit distinct differences between each control group.

For the experimental arms, studies generally incorporated all pathogenic variants into a *BRCA1*- or *BRCA2*-associated arm. However, Waddell et al. [13] observed differences between truncating and missense pathogenic variants. Mixed populations of variant types used by other studies may confound any observed difference. Furthermore, two studies highlighted that sporadic tumours with hypermethylated *BRCA1* promoters exhibit expression profiles similar to *BRCA1* pathogenic variants [6,8]. Two further studies directly addressed the hypermethylated *BRCA1* promoter by excluding [22] or combining with them pathogenic variants [20]. In addition, George et al., combined tumours with *BRCA1* and *BRCA2* somatic mutations with germline pathogenic variants. It remains unclear whether tumours that harbour somatic mutations would exhibit similar expression profiles to tumours developed in germline pathogenic variant carriers. In addition, one study had particularly extreme exclusion criteria for both control and experimental ovarian tumours [17]. Konstantinopoulos and colleagues selected only those tumours which clustered in the correct groups, effectively removing tumours in order to fit classifications. It is unlikely that this method of sample selection would generate a gene expression signature reflective of *BRCA1* or *BRCA2* carriers. This was highlighted by an independent study which demonstrated poor accuracy of the Konstantinopoulos “BRCA-like” signature [22].

Secondly, the importance of sample matching was highlighted by the early studies in breast tumours which identified ER status as a major driver of expression variation [8]. Subsequent studies showed that tumour subtype was also a major driver of variability in expression [18,19,25]. The development of ovarian subtypes is not yet as well defined as those in breast. However, Tothill et al. [34] identified six molecular subtypes in high-grade ovarian tumours. Subsequently, *BRCA1*-associated ovarian tumours were shown to be enriched for immunoreactive (C2) subtype, which is characterised by lymphocytic infiltrate in the epithelium [22]. However, these have yet to be a consideration in *BRCA*-associated gene expression studies in ovarian cancers. Despite inconsistencies in tumour matching between studies, the similarities between a small subset suggest that differences between study expression profiles are not solely due to sample matching. For example, three studies investigated *BRCA1*-associated expression profiles in ER-negative breast cancers [8,11,12] and no gene was common between these three studies (Table 2).

Third is the purity of the sample collected for RNA isolation and the influence of cell type heterogeneity (e.g., tumour and stromal cells) on gene expression profiles. Subsequent to the majority of studies Massink et al. [25] highlighted the importance of invading tumour lymphocytes on gene expression profiles. The presence of invading tumour lymphocytes, a feature of *BRCA1*-associated breast tumours, added complexity to gene expression profiles. Although the effect of lymphocyte presence was not tested, George et al., highlighted the enrichment of *BRCA1*-associated ovarian tumours for the C2 molecular subtype. The C2 subtype is characterised by the presence of lymphocytes in the epithelial fraction [34]. Four studies [8,18,23,31] selected samples based on >50% tumour content as assessed by histological review, and one study [16] enriched for tumour content using laser capture microdissection. The remaining studies digested tumour samples with no apparent knowledge of cellular content.

Taken together, there are subtle differences in sample selection criteria across all studies. It is difficult to determine the effect these differences have on recapturing gene expression changes. However, there were several consistent observations between studies, for example the ability to identify the ER status of tumours based on expression profiles [8,19]. Furthermore, there was consistent association of *BRCA1*-associated breast tumours with basal-like subtype [18,19,25]. As these observations were consistent, it can be assumed that any *BRCA1/2* gene expression profile is more subtle, and would be confounded by any differences in molecular features.

### 5.2. Differences in DNA Damaging Treatments of Normal Tissue

Table 3 summarises the differences in the design and methods of studies in normal tissue, which may limit their comparability. The majority of studies (6/10) induced DNA damage to elicit a change in expression, under the hypothesis that *BRCA1* and *BRCA2* carriers would have an impaired DNA damage response. Only Walker et al., used Mitomycin C as a DNA damage-inducing reagent as the authors observed a greater effect on expression compared to *γ*-irradiation [15]. The remaining five studies treated fibroblast [9,10], lymphocytes [21] and LCLs [13,24] with *γ*-irradiation. A further four studies [14,26,27,28] identified expression changes in untreated LCLs. Despite LCLs requiring Epstein–Barr virus (EBV) transformation to become immortal, there is evidence that transformation has little effect on gene expression profiles [38]. However, *BRCA1*-associated LCLs are not susceptible to DNA damage, a phenotype expected for functionally damaged BRCA1 [39]. Thus it is important to better understand the effect of EBV transformation in the context of impaired *BRCA1* to appreciate the impact on gene expression.

### 5.3. Advancement in Technologies and Statistical Approaches

Early gene expression studies were limited by both cost and microarray technology. The earliest studies used cDNA spotted arrays, which were limited to 1000 s of targets, while the more modern arrays are able to detect 10,000 s of targets and next generation sequencing platforms are able to detect the entire transcriptome. Such advancement in gene expression technology means that the latter studies are measuring transcripts not tested in the earlier studies [40,41]. Furthermore, transcript annotations and guidelines around publishing large gene expression array studies have developed alongside these studies. This led to occasions where early gene panels could not be validated in later, much larger expression arrays. For example Dudaladava et al., only had expression data for 40 of the Hedenfalk 51 gene panels, making validation of earlier results difficult [6,11]. The reduced cost of performing transcriptome analysis and ongoing importance of tissue biobanking has allowed more recent studies to test a greater number of samples thus increasing statistical power [18,23]. Importantly, genome-wide transcriptome analysis requires consideration of multiple testing to control the false positive rate. A total of 14 of the 23 studies discussed here used no *p*-value adjustments and 2 of the 23 studies [7,27] set a strict *p*-Value threshold (*p* < 0.0001). Eight of the remaining nine studies controlled the false-discovery rate, an attempt to reduce false positives, whilst the last used the more conservative Bonferroni correction method. Although *p*-values were not adjusted in the majority of studies, given sufficient power and effect size we would still expect that true positive results be shared between different expression datasets independent of the method and technology applied. However, only a small overlap of genes associated with *BRCA1/2* pathogenic variant status was observed between studies (Table 2).

### 5.4. Methods of Transcriptome Analysis

Two general approaches, class prediction and differential expression analysis, have been undertaken to identify gene differences between tumour genotypes. Class prediction attempts to identify genes with the ability to segregate samples into distinct subgroups, whilst differential expression identifies genes that differ between multiple subgroups. The latter approach requires knowledge of the genotypes or tumour subtypes. If the major driver of expression variability was due to the variant status of the tissue then it would be reasonable to expect a large overlap of gene lists between methodologies, as seen by Kote-Jarai et al. [9]. However, the aforementioned differences in study design and sample selection has likely led to study biases and confounding variables, which dilute any genotype-associated expression differences. Furthermore, the selection of genes lists was inconsistent. For example, studies using class prediction methods typically optimised gene lists to contain the smallest number of genes required to accurately segregate samples into subgroups. A common limitation between studies was the lack of validation in independent datasets. To be able to fully appreciate the strength of a classifier and to compare performance between studies these independent validations need to be performed.

## 6. Conclusions

Identifying individuals with pathogenic variants in *BRCA1* or *BRCA2* is critical in the management and prevention of breast and ovarian cancer. The increasing DNA profiling of tumours will continue to identify greater number of variants of uncertain significance and the need to classify these will become greater.

Due to the enormity of literature surrounding *BRCA1* and *BRCA2* pathogenic variants, there may further gene expression studies outside of this review. However, the inclusion of any further study would not materially change the conclusions, rather they would further highlight the complexity of *BRCA1*-associated gene expression phenotypes.

The studies included in this review have each identified gene sets associated with *BRCA1* or *BRCA2* variant status. Despite overlapping aims, there is a distinct lack of consensus between datasets (Table 4). Rather, each study identified specific genes likely driven by the differences in study designs rather than *BRCA1/2* variant status. The lack of consensus may be due to differences in study design and statistical approaches (Table 4). Furthermore, all studies had modest cohort sizes (<80 familial breast cancers) that limited the ability to identify subtle changes in expression.

It also remains unclear from the current studies whether somatic changes occurring during tumorigenesis overwhelm any germline “BRCA-like” expression profile. For tumours with no pathogenic variant, *BRCA1* promotor hypermethylation status should be determined as it is unclear whether this would lead to expression profiles that mimic those associated with pathogenic variants. Despite the lack of consensus between study datasets, gene expression profiles associated with pathogenic variant status remain as a potential molecular phenotype to aid in variant classification. However, utilising the advancements in expression-based technologies, developing larger study cohorts and better understanding the variables affecting gene expression profiles across different tissue types must be carefully considered for future studies.

## Figures and Tables

**Table 1:** Experimental approaches for gene expression studies of BRCA1/2 pathogenic variants BRCAx pathogenic variants or controls.

Reference	Tissue Source	Statistical Analysis	*P*-Value Adjustments	Number of Samples (Number of Familial Samples)	Transcriptome Platform
Hedenfalk et al. [6] (2001)	Breast tumour	LOO-CV, Modified F-test, t-test, weighted gene analysis and InfoScore	None	21 (7 *BRCA1,* 7 *BRCA2*)	cDNA array (~6500 probes)
Jazaeri et al. [7] (2002)	Ovarian tumour	Modified F-test	None, *p* < 0.001	61 (18 *BRCA1*, 16 *BRCA2*)	cDNA microarray chips (7651 probes)
van’t Veer et al. [8] (2002)	Breast tumour	Pearson correlation, LOO-CV	None	98 (18 *BRCA1*, 2 *BRCA2*)	Hu25K microarrays
Kote-Jarai et al. [9] (2004)	Breast fibroblasts	Class prediction, SAM, SVM, LOOCV,	None	14 (9 *BRCA1*)	cDNA array (~5600 probes)
Kote-Jarai et al. [10] (2006)	Breast fibroblasts	SVM, Fisher score, t test Mann-Whitney, GPC, LOO-CV.	None	30 (10 *BRCA1*, 10 *BRCA2*)	cDNA array (~14,000 probes)
Dudaladava et al. [11] (2006)	Breast tumour	Parametric Welch test	BH	20 (7 *BRCA1*)	Affymetrix HG U133 Plus 2.0 Gene Chip
Martin et al. [12] (2007)	Breast tumour	Random variance t tests	None	van’t Veer dataset	Hu25K microarrays
Waddell et al. [13] (2008)	LCLs	SVM, Fisher score, t test Mann-Whitney, GPC, LOO-CV.	None	72 (23 *BRCA1*, 22 *BRCA2*, 27 BRCAx)	Illumina Human-6 version 1 BeadChips
Vuillaume et al. [14] (2009)	Peripheral blood	Welch t-test	BH	30 (15 *BRCA1,* 15 BRCAx)	Agilent 44 K Whole Human genome Oligo Microarray
Walker et al. [15] (2010)	LCLs	F-test, t-test	FDR – Korn et al. approach	36 (9 *BRCA1,* 9 *BRCA2,* 9 BRCAx)	Illumina HumanRef8-V2 Beadchips
Press et al. [16] (2010)	Fallopian tube and ovarian tumour	Welch t-test	None, *p* < 0.01	30 (19 *BRCA1*)	Affymetrix HG U133A Plus 2.0 Gene Chip
Konstantinopoulos et al. [17] (2010)	Ovarian tumour	Fisher’s exact test	None	Jazaeria et al., (2002) 61 (18 *BRCA1*, 16 *BRCA2*)	cDNA microarray chips (7651 probes)
Waddell et al. [18] (2010)	Breast tumour	limma	None, positive B	75 (19 *BRCA1,* 30 *BRCA2,* 25 BRCAx)	Illumina Human-6 version 2 BeadChips
Lisowska [19] (2011)	Breast tumour	Welch t test, ANOVA	BH	35 (12 *BRCA1*, 1 *BRCA2,* 5 BRCAx)	Affymetrix HG U133 Plus 2.0 Gene Chip
Jönsson et al. [20] (2012)	Breast tumour	Fisher exact test	None	577 (34 *BRCA1*, 39 *BRCA2)*	Swegene H_v2.1.1 55K
Salmon et al. [21] (2013)	Lymphocytes	one-way Welch ANOVA	BH	80 (13 *BRCA1,* 10 *BRCA2*, 43 BRCAx)	Affymetrix U133A Plus 2.0
	Ovarian tumour	limma	FDR	TCGA dataset (27 *BRCA1*, 28 *BRCA2,* 145 BRCAx)	RNA-sequencing
George et al. [22] (2013)				AOCS dataset (18 *BRCA1*, 11 *BRCA2*, 103 BRCAx)	Affymetrix U133A Plus 2.0
				Jazaeria et al., (2002) 61 (18 *BRCA1*, 16 *BRCA2*)	cDNA microarray chips (7651 probes)
Larsen et al. [23] (2013)	Breast tumour	limma, Welch t-test, LOOCV, SVM	None	183 (33 *BRCA1, 22 BRCA2*)	Agilent SurePrint G3 Human GE 8 × 60K Microarray
Feilotter et al. [24] (2014)	LCLs	PAM, SAM	None	69 (31 *BRCA1*)	Agilent Whole Human Genome Oligo 4 × 44K GE arrays
Massink et al. [25] (2015)	Breast tumour	ANNOVA	FDR	Nagel et al., (2012) 120 (17 CHEK2, 35 BRCA1, 5 BRCA2, 63 BRCAx)	Affymetrix U133A Plus 2.0
Pouliot et al. [26] (2017)	LCLs	ANOVA, Scheffe post-hoc test	Bonferroni correction	117 (36 *BRCA1*, 49 *BRCA2,* 32 BRCAx)	RNA Sequencing
Zahavi et al. [27] (2018)	Lymphocytes	limma	None, *p* < 0.001	50 (11 *BRCA1*, 13 *BRCA2*)	mRNA Sequencing
Tanaka et al. [28] (2018)	Peripheral blood	ANOVA	None	40 (10 *BRCA1,* 10 *BRCA2*, 9 BRCAx)	Illumina DASL array
Akbari et al. [29] (2019)	Breast tumour	Four study Meta-analysis (Fisher’s). limma	FDR	45 (*BRCA1 BRCA2*) + 25 BRCAx)	Illumina Human-6 version 1 BeadChips
				43 (*BRCA1 BRCA2*) + 29 BRCAx)	Illumina Whole Genome-DASL
				55 (*BRCA1 BRCA2*) + 65 BRCAx)	Agilent SurePrint G3 Human GE 8x60K Microarray
				53 (*BRCA1 BRCA2*) + 76 BRCAx)	Affymetrix U133A Plus 2.0

BH, Benjamini-Hochberg adjustment. BRCAx; non-*BRCA1/2.* FDR; False discovery rate. GPC; Gaussian Process Classifier. LCLs; lymphoblastoid cell lines. LOO-CV; Leave One Out Cross Validation. PAM, Prediction Analysis of Microarray. SAM; Significance Analysis of Microarrays. SVM; Support Vector Machine.

**Table 2:** Gene overlap between gene expression profiles.

	Breast Tumour									Ovarian Tumour				Fibroblast		LCLs/Lymphocytes/Blood							
	Hedenfalk et al., 2001	van ’t Veer et al., 2002	Dudaladava et al., 2006	Martin et al., 2007	Waddell et al., 2010	Lisowska, 2011	Jönsson et al., 2012	Larsen et al., 2013	Akbari et al., 2013	Jazaeri et al., 2002	Press et al., 2008	Konstantinopoulos et al., 2010	George et al., 2013	Kote-Jarai et al., 2004	Kote-Jarai et al., 2006	Waddell et al., 2008	Vuillaume et al., 2009	Walker et al., 2010	Salmon et al., 2013	Feilotter et al., 2014	Pouliot et al., 2017	Tanaka et al., 2018	Zahavi et al., 2018
Hedenfalk et al. [6] 2001 (*n* = 50)		0	1	0	3	1	0	1	2	0	0	2	0	3	1	0	2	1	0	0	2	2	0
van ’t Veer et al. [8] 2002 (*n* = 71)			0	0	1	0	0	0	5	0	2	1	0	2	2	1	0	1	0	0	0	0	3
Dudaladava et al. [11] 2006 (*n* = 88)				0	1	9	0	2	0	0	0	0	0	1	2	1	1	4	0	0	2	0	1
Martin et al. [12] 2007 (*n* = 3)					0	0	0	0	1	0	0	0	0	1	0	0	0	0	0	0	0	0	0
Waddell et al. [18] 2010 (*n* = 227)						3	0	2	49	0	0	1	2	2	7	3	1	9	0	0	0	0	5
Lisowska, [19] 2011 (*n* = 61)							0	0	5	0	2	0	1	0	1	1	0	5	0	0	1	0	0
Jönsson et al. [20] 2012 (*n* = 4)								0	0	0	0	0	0	1	0	0	0	0	0	0	0	0	2
Larsen et al. [23] 2013 (*n* = 210)									8	0	0	1	2	2	3	2	1	7	0	0	0	0	2
Akbari et al. [29] 2018 (*n* = 278)										1	2	2	1	4	4	1	1	7	1	1	0	0	7
Jazaeri et al. [7] 2002 (*n* = 12)											0	2	1	0	0	0	0	0	0	0	0	0	0
Press et al. [16] 2008 (*n =* 33)												0	1	0	2	0	0	1	0	1	0	0	0
Konstantinopoulos et al. [17] 2010 (*n =* 60)													0	2	3	3	0	2	0	0	1	0	0
George et al. [22] 2013 (*n =* 75)														5	3	2	1	2	0	0	0	0	1
Kote-Jarai et al. [9] 2004 (*n =* 122)															3	2	2	4	0	1	1	1	3
Kote-Jarai et al. [10] 2006 (*n =* 330)																3	0	21	1	2	1	4	3
Waddell et al. [13] 2008 (*n =* 333)																	1	11	0	2	0	1	3
Vuillaume et al. [14] 2009 (*n =* 81)																		5	0	0	3	1	1
Walker et al. [15] 2010 (*n =* 687)																			1	4	3	3	3
Salmon et al. [21] 2013 (*n =* 21)																				1	0	0	0
Feilotter et al. [24] 2014 (*n =* 43)																					0	1	0
Pouliot et al. [26] 2017 (*n =* 62)																						1	4
Tanaka et al. [28] 2018 (*n =* 51)																							1
Zahavi et al. [27] 2018 (*n =* 228)																							

Black text, between tissue comparison; blue text across tissue comparison; (*n* = The number of unique gene symbol).

**Table 3:** A summary of design and methods within of studies focusing on normal tissue. BC-Breast cancer, OC-Ovarian cancer, LCLs-Lymphoblastoid cell lines.

Study (Year)	Tissue Source	Tissue Criteria	Comparison(s)	Treatment
Kote-Jarai et al. [9] (2004)	Breast fibroblast	Prophylactic mastectomy or breast reduction	*BRCA1* vs. non-*BRCA1*	γ irradiation
Kote-Jarai et al. [10] (2006)	Breast fibroblast	Skin biopsy women post BC (disease-free at time of collection)	*BRCA1* vs. BRCAx; *BRCA2* vs. BRCAx	γ irradiation
Waddell et al. [13] (2008)	LCLs	Breast cancer affected women	*BRCA1* (truncating variants) vs. BRCAx; *BRCA1* (missense variants) vs. BRCAx; BRCA2 (truncating variants) vs. BRCAx; BRCA2 (missense variants) vs. BRCAx;	γ irradiation
Vuillaume et al. [14] (2009)	Peripheral blood Mononuclear Cells	High risk breast cancer family members, disease-free at time of collection (some had BC/OC history)	*BRCA1* vs. non-*BRCA1*	None
Walker et al. [15] (2010)	LCLs–pooled from 3x individuals	Breast cancer affected women	*BRCA1* vs. *BRCA2* vs. BRCAx	Mitomycin C
Salmon et al. [21] (2013)	Lymphocytes	No personal history of cancer with family member with history of breast cancer	*BRCA1* vs. BRCAx; *BRCA2* vs. BRCAx	γ irradiation
Feilotter et al. [24] (2014)	LCLs	Samples deposited in NIH Breast Cancer Family Registries with and without *BRCA1* variants.	*BRCA1* vs. non-*BRCA1*	γ irradiation
Pouliot et al. [26] (2017)	LCLs	Individuals of high risk breast cancer families with and with disease history. Included the oldest BRCAx sister with no cancer history	*BRCA1* vs. BRCAx (no BC history); *BRCA2* vs. BRCAx (no BC history); BRCAx (BC history) vs. BRCAx (no BC history)	None
Tanaka et al. [28] (2018)	Peripheral blood	Carriers of pathogenic *BRCA1* or *BRCA2* variant with or without cancer, BRCAx carriers with cancer.	*BRCA1* vs. non-*BRCA1*; *BRCA2* vs. non-*BRCA2*	None
Zahavi et al. [27] (2018)	Lymphocytes	No personal history of cancer. Controls had no family history of BC or OC	*BRCA1* vs. *BRCAx; BRCA2* vs. *BRCAx*	None

**Table 4:** Difference in study design that limit comparability.

**Study design**
Variant classifications (missense/truncating, germline/somatic)
Sample size
Control arm (unselected, BRCAx, healthy controls)
Technology (spotted microarray, oligo array, RNA-sequencing)
Tissue type/purity
Tissue treatments
**Identification of *BRCA1* associated genes**
Class comparison
Class prediction
**Other**
Non-variant effect
Epigenetic silencing
Environmental effects
